# Primary Endometrial Lymphomas: A Systematic Review

**DOI:** 10.3390/diagnostics16060849

**Published:** 2026-03-12

**Authors:** Mahmoud Rezk Abdelwahed Hussein, Manal Bahkali, Toka Mahmoud R. A. Hussein, Eman Abu-Dief, Ahmed R. Abdulwahed

**Affiliations:** 1 Department of Pathology, Faculty of Medicine, Assiut University Hospitals, Assiut University, Assiut 71515, Egypt; 2 Department of Pathology, Jazan Military Hospital, Jazan 84224, Saudi Arabia; mnl.bahkali@gmail.com; 3 Faculty of Medicine, Sohag University, Sohag 82524, Egypt; dereer681@gmail.com; 4 Histology Department, Faculty of Medicine, Sohag University, Sohag 82524, Egypt; eman_elmadany@yahoo.com; 5 Department of Obstetrics and Gynecology, Faculty of Medicine, Al-Azhar University, Cairo 11651, Egypt; ahmedrezk801@yahoo.com

**Keywords:** primary, endometrium, lymphoma, immune cells, pathology

## Abstract

**Background:** Primary endometrial lymphomas (PELs) are exceedingly rare and diagnostically challenging lesions. **Objective:** To assess the clinicopathologic features of PELs. **Methods:** We adhered to the PRISMA-2020 guidelines for reporting systematic reviews. A PubMed literature search (1956–2025) was conducted using keyword combinations including “endometrium” and “lymphoma,” “lymphoid proliferation,” or “lymphoproliferative lesions.” Only original articles published in the English peer-reviewed journals were considered. The inclusion criteria were: (i) studies involving human subjects, and (ii) studies published in the English language. Reviews, editorials, meeting abstracts, and non-English publications were excluded. **Results:** We identified 42 studies for our analysis, collectively reporting 58 cases of PELs. Abnormal uterine bleeding was the main complaint. Non-Hodgkin lymphoma (57 cases) and Hodgkin lymphoma (one case) were identified. In most cases, lymphoma was the sole lesion. In five cases, lymphoma coexisted with, preceded, or followed endometrial carcinoma. Histologically, PELs either diffusely involved the endometrium (50 cases) or were localized to endometrial polyps (eight cases). Marginal zone lymphoma (MZL) was the most frequently reported type, followed by diffuse large B-cell lymphoma (DLBCL). Other rare types included intravascular large B-cell lymphoma, NK/T-cell lymphoma, T-cell lymphoma, and low-grade B-cell lymphoma. **Conclusions:** Our study indicates that MZL and DLBCL were the most common types of PELs. Other extremely rare subtypes were also identified. Moreover, some PELs developed in the background of endometrial polyps and, in exceptional cases, in association with endometrial carcinoma. Radiological findings were critical for provisional diagnosis, staging, and follow-up. Key modalities included ultrasonography (US), computed tomography (CT), magnetic resonance imaging (MRI), and 18F-fluoro-2-deoxyglucose positron emission tomography/CT (18F-FDG PET/CT).

## 1. Introduction

**Figure 1:**
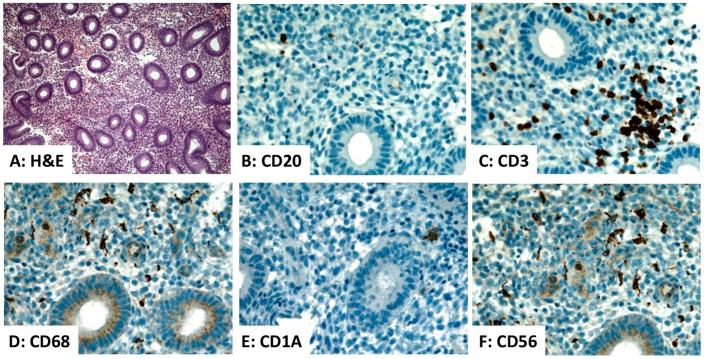
Distribution of immune cells within the proliferative phase endometrium. (**A**) Hematoxylin and eosin (H&E)-stained section of normal proliferative endometrium (×100). (**B**–**F**) Immunohistochemistry demonstrates rare CD20^+^ B lymphocytes, an aggregate of CD3^+^ T lymphocytes, scattered CD68^+^ histiocytes, rare CD1A^+^ dendritic cells, and scattered CD56^+^ natural killer cells within the endometrial stroma ((**B**–**F**), ×400). Source: Image is the own work of the corresponding author, Professor M. Hussein.

**Figure 2:**
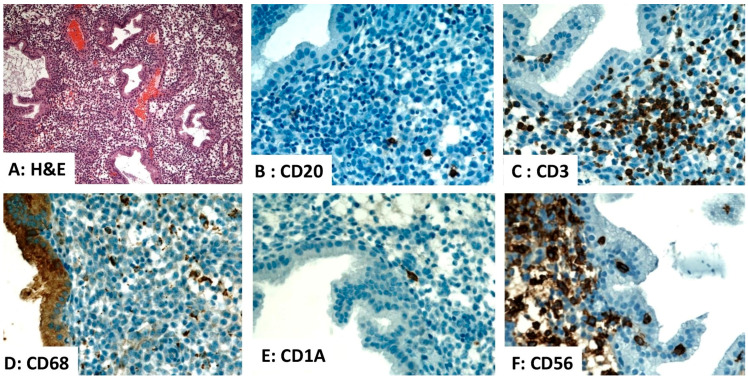
Distribution of immune cells within the secretory phase endometrium. (**A**) Hematoxylin and eosin (H&E)-stained section of normal secretory endometrium (×100). (**B**–**F**) Immunohistochemistry demonstrates rare CD20^+^ B lymphocytes, an aggregate of CD3^+^ T lymphocytes, numerous CD68^+^ histiocytes, rare CD1A^+^ dendritic cells, and abundant CD56^+^ natural killer cells within the endometrial stroma ((**B**–**F**), ×400). Source: Image is the own work of the corresponding author, Professor M. Hussein.

The endometrium harbors several types of immune cells with distributions and densities that vary across the phases of the menstrual cycle [1]. These include lymphocytes, histiocytes, natural killer (NK) cells, and dendritic cells (DCs), which play essential roles in immune surveillance, embryo implantation, and endometrial homeostasis. Leukocytes constitute approximately 6–20% of the total stromal cell population, with T lymphocytes representing 30–60% of that leukocyte population [2]. A marked decrease in leukocyte density occurs in the postmenopausal endometrium resulting from the decline in hormonal signaling [2]. A summary of the lymphoid cells and DCs in the normal human endometrium is presented in Table 1 and Figure 1 and Figure 2.

### 1.1. Endometrial Lymphocytes, Natural Killer Cells, Histiocytes, and Dendritic Cells

T lymphocytes are the predominant endometrial lymphoid cell population, consisting primarily of CD8^+^ cytotoxic T cells with a small number CD4^+^ helper T cells [1,16]. Their functions include immune surveillance, modulation of inflammation, and defense against microbial pathogens. Endometrial B lymphocytes are sparse, representing less than 5% of the total endometrial immune cells. Their numbers increase significantly in cases of chronic endometritis and infertility associated with autoimmunity [2,17] (Figure 1 and Figure 2). Uterine natural killer cells (uNK) are a non-cytotoxic subset of lymphoid cells. Their numbers increase markedly during the secretory phase. uNK cells are essential for the establishment of pregnancy [1,9]. The distribution of these immune cells in the human endometrium is depicted in Figure 1 and Figure 2.

Histiocytes (macrophages) are phagocytic cells that play crucial roles in angiogenesis, tissue repair, and remodeling following endometrial shedding, as well as in antigen presentation and modulation of the local immune response [11,18]. They account for 1–2% of stromal cells during the proliferative phase, increase to 3–5% during the secretory phase, and peak at 6–15% during menstruation [8,19]. Endometrial DCs comprise approximately 2% of decidual leukocytes. They play pivotal roles in antigen presentation, T-cell modulation, and the establishment of immune tolerance at the maternal–fetal interface [13,20]. The distribution of these immune cells in the human endometrium is depicted in Figure 1 and Figure 2.

### 1.2. Endometrial Lymphoid Aggregates

In the human endometrium, there are three distinct zones of lymphoid tissue: intraepithelial, interstitial, and aggregated forms within the basal layer, known as endometrial lymphoid aggregates (ELAs) [14]. These zones are primarily composed of T cells, with rare B cells and DCs [14]. ELAs develop through the continuous recruitment of circulating immune cells. They consist of a central core of B lymphocytes surrounded by CD8^+^ T lymphocytes and an outermost layer rich in histiocytes (macrophages) [21]. The development of ELAs is hormonally regulated. They are rarely observed during the early proliferative phase but become more apparent during the secretory phase and typically disappear after menopause [21]. Furthermore, some endometrial immune cells undergo local proliferation throughout each menstrual cycle [22].

### 1.3. Endometrial Lymphoproliferative Lesions

The vast majority of endometrial lymphomas represent secondary involvement of the endometrium due to the systemic dissemination of lymphoma originating elsewhere. The endometrial lymphoproliferative lesions encompass a spectrum of reactive (lymphoma-like lesions) and neoplastic conditions (primary endometrial lymphomas, PELs). PELs are exceedingly rare malignancies that are often missed because their clinical presentations involve abnormal vaginal bleeding or irregular cycles, which leads physicians to suspect more common conditions such as dysfunctional uterine bleeding or other neoplasms (leiomyomas and carcinomas).

**Figure 3:**
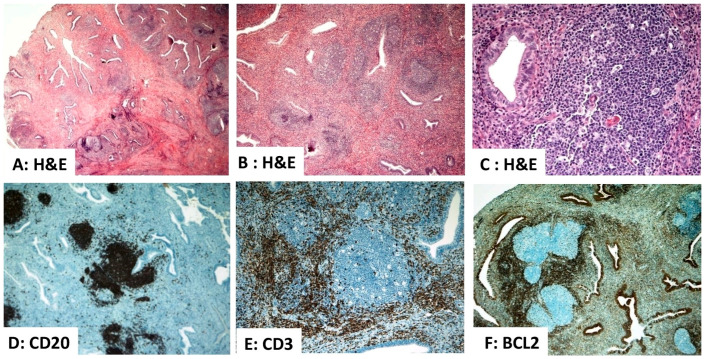
Lymphoid hyperplasia arising within an endometrial polyp. (**A**–**C**) Hematoxylin and eosin (H&E)-stained sections demonstrate stromal expansion by dense, nodular lymphoid infiltrates ((**A**) ×20). The architecture includes well-formed reactive follicles with prominent germinal centers containing tingible body macrophages and preserved mantle zones ((**B**) ×40). The germinal center is composed of lymphocytes and tingible body macrophages, whereas the mantle zone consists of small, mature lymphocytes ((**C**) ×200). (**D**–**F**) Immunohistochemistry demonstrates strong reactivity for B lymphocytes (CD20^+^, (**D**) ×40) in their expected localization. CD3 staining highlights lymphocytes in the mantle zone and interfollicular areas ((**E**) ×100), confirming a mixed population of B and T cells. Negative BCL2 staining in the germinal centers supports the reactive nature of the lymphoid infiltrates ((**F**) ×100). Source: Image is the own work of the corresponding author, Professor M. Hussein.

The endometrial lymphoma-like lesions are florid, reactive lymphoid proliferations that are typically associated with chronic antigenic stimulation or hormonal changes. These lesions can sometimes mimic malignant lymphomas due to their high cellularity. There are several lymphoma-like lesions that can mimic PELs. They include chronic endometritis (with a prominent lymphoid infiltrate), hyperplastic ELAs, extensive aggregates of endometrial granulocytes (uNK cells), endometrial polyps with extensive nodular or diffuse reactive lymphoid infiltrates, and infection-associated lymphoid hyperplasia including infectious mononucleosis and cytomegalovirus [21,23,24,25,26]. Chronic endometritis is a diffuse inflammatory process, while a benign polyp with a dense lymphoid infiltrate represents a localized, mass-forming reactive proliferation. Although chronic endometritis and polyps are reactive, their clinical and pathological presentations differ. Key features that separate these reactive conditions from PELs include the presence of a polymorphous cell population, a lack of significant cytologic atypia or mitotic activity in the large cells, and a polyclonal B-cell population (no light-chain restriction). Immunohistochemistry and molecular studies are important ancillary techniques that help establish the reactive nature of these lymphoid endometrial lesions. A case of lymphoid hyperplasia arising within an endometrial polyp is depicted in Figure 3.

### 1.4. Primary Endometrial Lymphomas

PELs are exceptionally rare gynecologic malignancies. They represent less than 1% of extranodal non-Hodgkin lymphomas (NHLs). There is no precise global prevalence rate for PELs in the general population due to their rarity. Available prevalence data fall under the general categories of female genital tract cancer, uterine cancer, and extranodal lymphomas. Primary NHL of the female genital tract represents 0.2% to 1.1% of all extranodal primary lymphomas [27,28,29]. The vast majority of PELs are marginal zone lymphoma (PE-MZL) and diffuse large B-cell lymphoma (PE-DLBCL). Other rare types include PE intravascular large B-cell lymphoma (PE-IVLCL), NK/T-cell lymphoma (PE-NKTL), T-cell lymphoma, (PE-TCL) and low-grade B-cell lymphoma. Accurate diagnosis of PELs is critical due to their clinical and histopathologic overlap with more common uterine neoplasms, including stromal sarcoma [30], undifferentiated sarcoma, and undifferentiated carcinoma.

#### 1.4.1. Primary Endometrial Marginal Zone Lymphoma

PE-MZL is a rare and indolent malignancy that usually affects postmenopausal women. It generally mirrors MZL of other organs at the immunohistochemical and molecular levels. The tumor cells express pan-B-cell markers CD19, CD20, CD22, and CD79a, as well as surface immunoglobulins—typically IgM with light-chain restriction. The neoplastic cells are negative for CD5, CD10, CD23, and cyclin D1. Expression of BCL2 is also commonly observed, while BCL6 and MUM1/IRF4 are generally absent or only weakly expressed [31,32].

Only a few cases of PE-MZL have been documented [31,32,33]. Tahmasebi et al. presented the largest case series to date (*n* = 4), where the median age of the patients was 59 years. Three women presented with abnormal uterine bleeding (AUB), while one case was discovered incidentally. None of the patients had evidence of lymphoma at other sites. Histological examination showed nodular endometrial proliferation of small lymphocytes with occasional immunoblasts and plasma cells. Immunophenotyping confirmed the diagnosis, and all cases demonstrated clonal immunoglobulin heavy chain gene rearrangement. Following a median follow-up period of three years, three of the patients remained alive and free of disease [31,32,33].

The differential diagnosis of PE-MZL includes chronic endometritis (with prominent lymphoid infiltrates), hyperplastic ELAs [14], extensive aggregates of endometrial granulocytes (uNK cells), endometrial polyps with extensive nodular or diffuse reactive lymphoid infiltrates, and infection-associated lymphoid hyperplasia, including infectious mononucleosis and cytomegalovirus [21,23,24,25,26].

#### 1.4.2. Primary Endometrial Diffuse Large B-Cell Lymphoma

**Figure 4:**
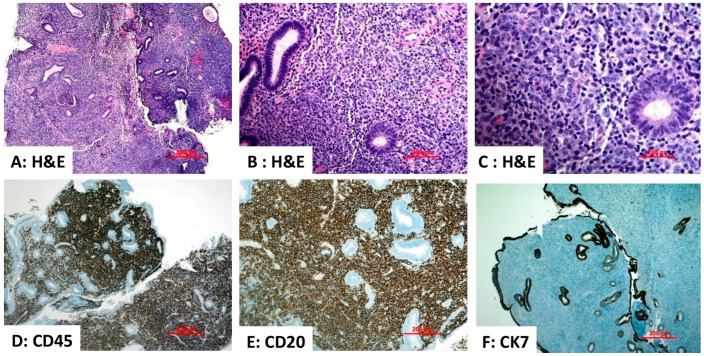
Primary endometrial diffuse large B-cell lymphoma. (**A**–**C**) Hematoxylin and eosin (H&E)-stained sections demonstrate diffuse effacement of the endometrial stroma by a monomorphic population of malignant lymphoid cells ((**A**) ×40). The neoplastic cells are medium to large in size ((**B**) ×200 and (**C**) ×400). (**D**,**E**) The neoplastic cells demonstrate strong positivity for the B-cell markers CD45 ((**D**) ×40) and CD20 ((**E**) ×400). (**F**) Staining for the epithelial marker cytokeratin 7 (CK7) is negative in the tumor cells, excluding the possibility of carcinoma ((**F**) ×40). Source: Image is the own work of the corresponding author, Professor M. Hussein.

Rare cases of primary endometrial diffuse large B-cell lymphoma (PE-DLBCL) have been reported, consistent with the typical paucity of B lymphocytes in endometrial tissue [34,35,36]. PE-DLBCL generally mirrors nodal DLBCL. The tumor cells express pan-B-cell markers such as CD20, CD79a, and PAX5. MUM1/IRF4 and BCL6 are typically expressed, whereas CD10 and BCL2 expressions occur in about half of all cases. Genetic rearrangements of BCL6, BCL2, and MYC are invariably observed, and this neoplasm is often classified as a non-germinal-center B-cell subtype per the Hans algorithm [34,37]. A case of PE-DLBCL is depicted in Figure 4.

Arshad et al. reported a rare case in a 49-year-old postmenopausal woman, which was initially suspected following the incidental finding of atypical cells on a Pap smear. Imaging revealed a hypermetabolic uterine mass, and the diagnosis was confirmed via biopsy. The patient attained complete remission following R-CHOP chemotherapy [34]. Cahill et al. described two cases in young, Black women with obesity who presented with abnormal vaginal bleeding; endometrial curettage and immunohistochemistry confirmed the diagnosis [38].

The differential diagnosis of PE-DLBCL is broad. It encompasses other PELs including Burkitt lymphoma (BL) [39,40], plasmablastic lymphoma (PBL), anaplastic large cell lymphoma (ALCL), extranodal NK/T-cell lymphoma, and myeloid sarcoma. This list also includes poorly differentiated non-lymphoid malignancies such as malignant melanoma, endometrial stromal sarcoma [30], undifferentiated/dedifferentiated endometrial carcinoma, and neuroendocrine carcinoma.

#### 1.4.3. Primary Endometrial Follicular Lymphoma

Primary endometrial follicular lymphomas (PE-FLs) are exceedingly rare tumors. Immunophenotypically and molecularly, PE-FLs mirror their nodal counterparts. The neoplastic cells usually exhibit a germinal-center B-cell immunophenotype, characterized by strong positivity for CD20, CD10, BCL2, BCL6, and PAX5, with most cases harboring the characteristic t(14;18)(q32;q21)/IGH–BCL2 translocation [41,42].

Miura et al. (2018) documented a rare case in a 48-year-old woman presenting with AUB and pelvic pain [42]. Histological examination of the hysterectomy specimen confirmed the diagnosis of PE-FL [42]. The differential diagnosis of PE-FL includes follicular lymphoid hyperplasia, hyperplastic nodular aggregates of ELAs [14], PE-MZL with a nodular growth pattern, mantle cell lymphoma (nodular pattern), small lymphocytic lymphoma/chronic lymphocytic leukemia with proliferation centers, and endometrial stromal nodule or low-grade endometrial stromal sarcoma [30].

#### 1.4.4. Primary Endometrial Intravascular Large B-Cell Lymphoma

IVLBCL is a rare form of extranodal lymphoma. It is characterized by the restricted proliferation of malignant B cells within the lumina of small and medium-sized blood vessels. This leads to microvascular occlusion and potentially fatal multiorgan dysfunction [43]. The homing of malignant large B cells to the vascular lumina results from defective extravasation, attributed to the loss of key adhesion molecules, which impairs transendothelial migration [44,45]. Immunophenotypically, IVLBCL cells display a mature B-cell phenotype, expressing CD20, CD19, CD79a, PAX5, and CD45. BCL2 and MUM1/IRF4immunostains are usually positive [43]. CD5 is observed in approximately 22–38% of cases, while CD10 and BCL6 are positive only in a minority. The Ki-67 proliferation index is typically high (>50–60%) [43].

Rare cases of PE-IVLBCL have been reported [46,47,48]. Diagnosis is challenging due to non-specific symptoms. Fujiwara et al. documented a case in a 62-year-old woman who presented with a fever of unknown origin. Endometrial curettage suggested a neoplastic process, and the examination of hysterectomy specimens revealed widespread IVLBCL confined mostly to vascular lumina [46]. Takeoka et al. reported a case in a 47-year-old female who presented with fever and anemia. Imaging revealed abnormal metabolic activity in the uterus, and an endometrial biopsy confirmed PE-IVLBCL. The patient received chemotherapy and remained in complete remission for 33 months post-treatment [47]. The differential diagnosis of PE-IVLBCL includes other PE-NHL (e.g., PE-DLBCL, and BL) [34,39,40,49,50] and metastatic carcinoma.

#### 1.4.5. Primary Endometrial Extranodal NK/T-Cell Lymphoma

ENKTLs are highly aggressive malignancies, typically diagnosed as the nasal type. PE-ENKTLs are exceedingly rare tumors [9]. Mehes et al. reported a case of PE-ENKTL in a 48-year-old woman who presented with abnormal vaginal bleeding. Endometrial biopsy revealed a dense, angiocentric, and angiodestructive lymphomatous infiltrate with areas of necrosis [49]. The tumor cells were positive for CD3ε, CD56, TIA-1, and granzyme B, and negative for CD5, CD4, CD8, and TCRγδ. Epstein–Barr virus (EBV) was detected by EBER in situ hybridization. The lymphomatous infiltrate was initially confined to the uterus but later disseminated rapidly despite intensive chemotherapy; consequently, the patient died within five months [49].

Wei et al. reported a case diagnosed by endometrial curettage. The patient underwent induction chemotherapy, but the disease progressed aggressively with bone marrow infiltration, and the patient died 76 days after diagnosis [50]. These findings emphasize the aggressive behavior of PE-ENKTL and the need for accurate and timely diagnosis [49,50].

The differential diagnosis of PE-ENKTL includes the florid physiological aggregates of uNK (CD56+) cells, chronic endometritis (lymphocyte rich), other PELs (such as peripheral T-cell lymphoma, and γδ T-cell lymphoma), and myeloid sarcoma. Additionally, the differential diagnosis includes small cell neuroendocrine carcinoma, melanoma, endometrial sarcoma, and inflammatory mimics with extensive necrosis [30].

#### 1.4.6. Primary Endometrial T-Cell Lymphoma

Primary endometrial peripheral T-cell lymphoma (PE-PTCL) is an exceedingly rare malignancy [51,52]. Immunophenotypically, the tumor cells express CD2, CD3, and CD5, with the loss of CD7. Murdoch et al. presented a case in which radiological workup revealed no evidence of disease outside the uterus. The patient underwent a hysterectomy followed by chemotherapy and remained disease-free 33 months post-treatment [51].

Lyman et al. described a rare relapse of precursor T-cell acute lymphoblastic leukemia/lymphoma in a 38-year-old woman, involving the uterine cervix, myometrium, endometrium, and appendix. The patient had been in remission for four years. Histologic examination revealed malignant lymphoid blasts infiltrating the endometrial stroma while sparing the glandular epithelium. Chemotherapy was reinitiated; however, the disease was refractory, and the patient died 10 months after the relapse [52]. The differential diagnosis of PE-PTCL includes other PELs (e.g., PE-DLBCL [34], BL [39,40], and NK/T-cell lymphoma) [49,50], leukemic infiltration, stromal sarcoma and high-grade carcinoma, and melanoma [30].

#### 1.4.7. Primary Endometrial Lymphoma Arising in a Background of Endometrial Polyps or Associated with Endometrial Carcinoma

**Figure 5:**
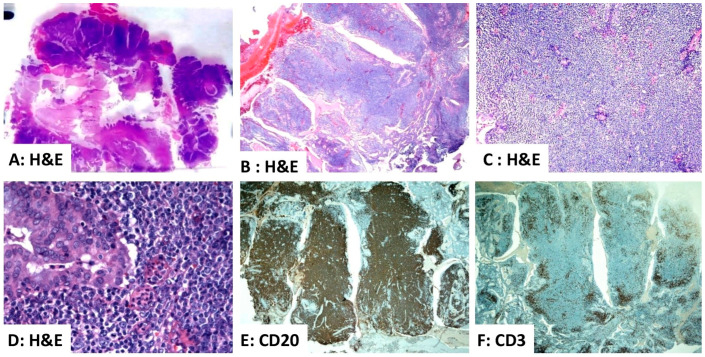
Primary endometrial diffuse large B-cell lymphoma arising within an endometrial polyp. (**A**–**D**) Hematoxylin and eosin (H&E)-stained sections show an endometrial polyp composed of a diffuse, dense lymphoid infiltrate of malignant lymphocytes ((**A**) ×20; (**B**) ×40; and (**C**) ×400). (**D**,**E**) Immunohistochemistry for CD20 demonstrates strong, diffuse positivity in the neoplastic cells ((**D**) ×20). (**F**) Staining for CD3 highlights scattered non-neoplastic, reactive T cells ((**F**) ×20). Source: Image is the own work of the corresponding author, Professor M. Hussein.

Several cases of PELs have been reported arising in the background of endometrial polyps [34,36,48,53,54,55,56]. Lemos et al. described the case of an 89-year-old woman with postmenopausal bleeding. A polypectomy was performed and immunohistochemical assessment revealed PE-DLBCL. The patient underwent hysterectomy but received no further treatment and died five months following diagnosis [54]. Scrimin et al. reported on a woman with postmenopausal vaginal bleeding resulting from intrauterine polyps. A polypectomy was performed and further histological examination revealed the presence of PE-MZL [56]. A case of PE-DLBCL arising in the background of an endometrial polyp is depicted in Figure 5.

### 1.5. Radiological Findings in PELs

Although the definitive diagnosis of PELs requires biopsy, radiological findings are important for provisional diagnosis, staging, and patient follow-up. The imaging modalities include ultrasonography (US), computed tomography (CT) and magnetic resonance imaging (MRI). These modalities can help assess the size, location, and extension of PELs. Moreover, Gallium-67 scintigraphy, 18F-fluoro-2-deoxyglucose positron emission tomography/computed tomography (FDG-PET/CT) (18F-FDG PET), and PET-CT can detect cases that may be overlooked by US, CT or MRI. 18F-FDG PET can also detect recurrence and predict the response to chemotherapy 18F-FDG PET [34,47,57,58,59,60].

Magnetic resonance imaging (MRI) is a valuable tool for diagnosis of PELs. It can reveal key features such as homogeneous signal intensity on T1 and T2 sequences, diffuse uterine enlargement, and a multinodular growth pattern [57,58,59]. Isosaka (2013) described a case of PE-DLBCL presenting as a bulky uterine tumor that was homogeneously low on T1-weighted imaging and isointense on T2-weighted imaging [57]. In a comparative study, Sugimoto (2013) found that PEL was characterized by diffuse uterine enlargement with a multinodular shape [58]. There was absence of endometrial or cervical epithelial thickening [58]. The apparent diffusion coefficient (ADC) values were very low due to restricted diffusion [58]. Similarly, advanced MRI techniques including T2-weighted imaging, contrast-enhanced scanning, diffusion-weighted imaging (DWI), and ADC mapping were used to evaluate PE-DLBCL of the uterine body [61].

18F-FDG PET can play an important role in the management of PELs. Okuda (2015) indicated the valuable utility of FDG-PET/CT in diagnosing two cases PE-DLBCL where ultrasound and MRI had revealed a normal-sized uterus and normal endometrium [60]. In both instances, FDG-PET/CT revealed abnormal FDG accumulation in the uterine body, leading to the diagnosis of lymphoma [60]. Takeoka (2011) reported a case of PE-IVLBCL involving the uterus, where CT and MRI revealed only a benign leiomyoma [47]. Alternatively, FDG-PET/CT showed FDG accumulation in the uterus, which successfully guided the biopsy site [47].

Computed tomography (CT) is less specific than MRI or PET-CT for characterizing the PELs. Isosaka (2013) used CT to demonstrate a pelvic DLBCL invading the retroperitoneum and causing bilateral hydronephrosis [57]. The absence of lymph node enlargement supported the diagnosis of PEL [57]. Ultrasonography (US) was often the initial imaging modality employed for diagnosis of PELs in patients presenting with abnormal uterine bleeding [60,61]. US frequently demonstrated a normal-sized uterus and normal endometrium, potentially leading to false-negative results [60]. Therefore, the primary limitation of US was that it may entirely overlook PELs [60]. This underscores the need for more advanced imaging such as MRI or PET-CT when clinical suspicion of PELs remains high [60].

Some cases of PELs have coexisted with endometrial carcinomas [35,62]. The proposed underlying mechanisms for the coexistence of these malignancies include a common genetic predisposition and cancer-related immunosuppression [63,64]. Vang et al. reported three patients diagnosed with both PEL and uterine carcinoma, including cases of synchronous and metachronous disease [62].

### 1.6. Knowledge Gap and Study Objective

The majority of endometrial lymphomas represent secondary involvement from systemic disease. PELs are exceptionally rare, and knowledge regarding their comprehensive clinicopathologic spectrum remains fragmented, largely confined to isolated case reports. To date, a synthesized analysis of all reported subtypes, their presentation, and associations (e.g., with polyps or carcinoma) is lacking. We conducted this systematic review to collate and analyze all published cases of PELs, aiming to provide a consolidated overview of their clinical presentations, histologic subtypes, diagnostic challenges, radiological features, and associated endometrial findings.

## 2. Materials and Methods

### 2.1. Protocol

We followed the PRISMA 2020 guidelines (the Preferred Reporting Items for Systematic Reviews and Meta-Analyses) from Supplementary Materials [65]. The review protocol has been registered with PROSPERO and is available at https://www.crd.york.ac.uk/PROSPERO/view/CRD420251253315 (accessed on 8 March 2026). The registration number for this review is CRD420251253315.

### 2.2. Search Strategy

A literature search was conducted using the PubMed database to examine the clinicopathologic features of PELs. This study analyzed previously published data and did not involve direct interaction with human subjects or access to individual medical records. Therefore, an institutional review board approval was not required. The research adhered to the ethical principles of the Declaration of Helsinki [66].

The review focused on relevant full-length case series and case reports published in international peer-reviewed journals. The PubMed (National Library of Medicine) database was searched for case reports and case series of PELs. The search strategy utilized the following keywords in the title/abstract fields: “endometrium” and “lymphoma,” “lymphoid proliferation,” or “lymphoproliferative lesions.”

### 2.3. Selection Criteria and Data Extraction

**Figure 6:**
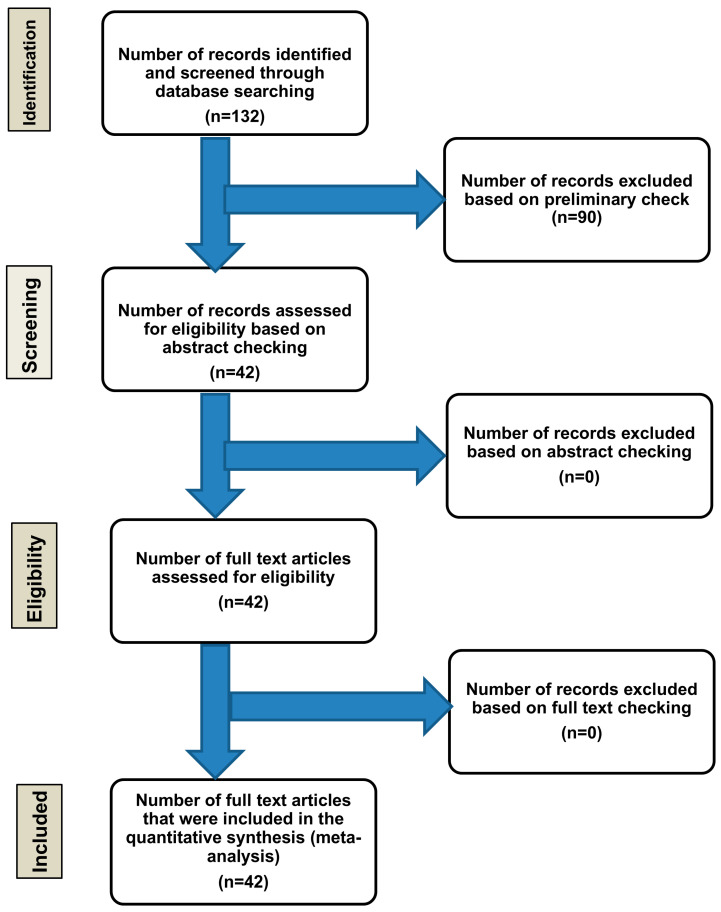
Study selection flowchart. A total of 132 studies were identified through the systematic review, of which 90 were excluded based on the predefined criteria. The remaining 42 studies met the inclusion criteria and were published between 1956 and July 2025, collectively reporting 58 cases of PELs. Source: Image is the own work of the corresponding author, Professor M. Hussein.

We first evaluated the eligible studies based on their titles, abstracts, and publication dates. Full texts were reviewed to verify eligibility. The authors independently screened all eligible studies. The preliminary exclusion criteria included: studies involving non-human subjects, clearly irrelevant titles, and studies published in languages other than English. To be included, studies had to: (i) involve human subjects, and (ii) be published in the English language while containing the search keywords. Studies that did not satisfy both conditions were excluded. Other publications, including reviews, editorials, and meeting abstracts were also excluded. Of the 132 articles identified by our search, 42 met the inclusion criteria and were included in the final analysis. The flowchart of the systematic review is shown in Figure 6. Due to the exceptional rarity of PELs, a comprehensive systematic narrative review necessitates the inclusion of seminal case reports and series published over several decades. These studies form the core of the literature. This approach preserves the integrity of the systematic search and accurately represents the literature for this entity.

### 2.4. Assessment of Study Quality and Risk of Bias

We used the Critical Appraisal Skills Programme (CASP) checklist to appraise the methodological quality of each article and the potential for bias. We chose this tool because it provides a clear structure suitable for evaluating case reports and case series [67]. As this is a systematic narrative review of case reports and series rather than a meta-analysis of comparative trials, a formal statistical quantitative bias assessment was not performed.

### 2.5. Data Synthesis

The goal of this review was to present a comprehensive descriptive synthesis of the current literature on the clinicopathologic features of PELs. The synthesis was narrative and descriptive. Two authors independently reviewed and interpreted all eligible studies. Discrepancies were resolved through discussion with a third author. The extracted variables, including patient age, clinical presentation, histologic subtype of lymphoma, and any associated cervical or endometrial pathologies, were grouped, organized, and synthesized into Table 2, Table 3, Table 4 and Table 5. Descriptive statistics were used to summarize the clinicopathologic features of the cases. Statistical analysis was performed using the IBM SPSS Statistics software, version 21.0 (IBM Corp., Armonk, NY, USA).

## 3. Results

A total of 132 studies were identified through the systematic review, of which 90 were excluded based on the predetermined criteria. The remaining 42 studies, published over a span of 69 years between 1956 to July 2025, met the inclusion criteria and collectively reported 58 cases of primary endometrial lymphoma. Of the 42 studies reviewed, all clearly outlined their case ascertainment approaches (including full clinical examination and radiological evaluation). The diagnoses in the included studies were established via histopathology. In older case reports, diagnosis relied solely on morphology. In more recent cases, the diagnosis was confirmed and subtyped using immunohistochemistry with molecular studies. The summaries of these cases are presented in Table 2, Table 3, Table 4 and Table 5.

### 3.1. Pathologic Features of PELs

All identified cases represented PELs. In 55 cases, the lymphoma was the only neoplastic lesion observed. In the remaining three cases, the lymphoma was associated with endometrial carcinoma. Specifically, three cases presented concurrently with endometrial carcinoma, one case preceded the subsequent carcinoma, and one case was followed by the development of subsequent carcinoma. The summaries of these cases are presented in Table 2, Table 3, Table 4 and Table 5.

### 3.2. Clinical Features and Diagnosis of PELs

The most common presenting symptom was AUB. The diagnosis was established primarily through endometrial biopsy. Immunohistochemical analysis was essential in all cases for confirming the diagnosis and subtyping the lymphoma.

### 3.3. Radiological Features and Diagnosis of PELs

Radiological evaluation (US, CT, MRI, and/or PET-CT) was performed in PELs for diagnosis, staging, and patient follow-up. The findings of imaging could not reliably distinguish PELs from more common entities such as carcinoma or leiomyoma. Therefore, the final diagnosis relied on the biopsy results [34,47,57,58,59,60].

MRI was valuable for characterizing PELs by revealing key features such as homogeneous signal intensity on T1 and T2 sequences, diffuse uterine enlargement, and a multinodular growth pattern [57,58,59]. A critical diagnostic clue was the preservation of the overlying endometrium despite extensive underlying stromal involvement, which helps differentiate lymphoma from carcinomas. Furthermore, MRI with DWI and low ADC values reflected the high cellularity of lymphomas [58].

In PELs, FDG-PET/CT served as a critical diagnostic tool, particularly in cases where conventional imaging (US, CT, or MRI) appeared normal or non-specific, by revealing intense FDG avidity in lymphomatous lesions of the uterus. It was invaluable for guiding biopsy to establish a tissue diagnosis, especially in challenging entities such as IVLBCL which lack solid tumors. Moreover, PET/CT played an essential role in disease staging. This modality can rule out nodal involvement and confirm primary uterine disease. Also, FDG-PET/CT can assess treatment response by confirming complete remission after chemotherapy [34,47,60].

CT was less specific than MRI or PET-CT for characterizing the PELs. It was primarily utilized for staging and detecting distant disease by revealing lymphadenopathy in extrauterine locations such as the mesentery or para-aortic region. CT was also useful for demonstrating the extent of local tumor burden, including invasion into the retroperitoneum and the assessment of complications like hydronephrosis caused by ureteral obstruction [57,59].

### 3.4. Histological Types of PELs

Histopathological evaluation showed that lymphomatous infiltration was diffuse throughout the endometrium in 50 cases, whereas in the remaining eight cases (mean age: 60.25 ± 4.90 years), the tumor cells were localized within endometrial polyps.

PE-MZL was the most frequently reported subtype, accounting for 22 cases (mean age: 59.33 ± 2.08 years) (Table 2). PE-DLBCL was the second most common PELs, observed in 20 cases (mean age: 53.05 ± 3.50 years) (Table 3). PE-IVLBCL accounted for four cases (mean age: 51.75 ± 7.75 years). PE-NKTL (mean age: 44.00 ± 2.31 years), PE-PTCL, and low-grade B-cell lymphoma were each reported in three cases, while BL was noted in two cases. A single case of Hodgkin lymphoma was also identified. Radiological and ultrasonographic imaging were performed in all cases to aid in diagnosis and staging. A summary of these cases is presented in Table 4.

### 3.5. Cases of Endometrial Lymphoma Presenting Within Endometrial Polyps or Coexisting with Endometrial Carcinoma

Our analysis identified eight cases (13.8%) presenting as lymphomas arising within an endometrial polyp (Table 5), including four PE-DLBCL, two PE-MZL, and two PE-DLBCL. The majority of cases occurred in postmenopausal women, while only two cases involved premenopausal or perimenopausal women (one case each). The mean age at diagnosis was 60.25 ± 4.9 years. Clinically, most patients presented with irregular vaginal spotting or abnormal vaginal bleeding. One case was detected incidentally. The diagnosis in all cases was confirmed through histopathological examination and immunohistochemical analysis. The adjacent endometrium may show disordered proliferative changes, weakly proliferative or simple atrophy. A detailed summary of the clinicopathological features of these cases is provided in Table 5.

Our study identified five published cases of endometrial NHL that occurred concurrently with, preceded, or followed a diagnosis of endometrial carcinoma. Among these, three cases were classified as PE-DLBCL, while one case each showed involvement by PE-FL and CLL/SLL. The mean age at diagnosis was 70.33 ± 9.39 years. Vaginal bleeding was the presenting symptom in all cases. The diagnoses were established based on a combination of radiological findings and immunohistochemical analyses.

## 4. Discussion

The mechanisms underlying the development of PELs are poorly understood; however, their pathogenesis may be linked to chronic inflammatory conditions such as chronic endometritis. Persistent antigenic stimulation can lead to the continuous recruitment, activation, and clonal expansion of B lymphocytes, potentially resulting in the establishment of ectopic lymphoid structures (ectopic tertiary lymphoid structures). This niche allows B lymphocytes to undergo somatic mutations, clonal expansion, and malignant transformation [17,31,87,88], resembling the development of MZL in other mucosa-associated lymphoid tissue [89].

The development of PE-ENKTLs may be linked to the retention of uNK cells, which can occur in conditions like uterine adenomyosis due to disruption of the normal cyclical shedding of the endometrium. Such retention potentially increases the risk of somatic mutations and malignant transformation [90]. Several mechanisms appear to contribute to the development of PE-IVLBCL, including immune evasion (e.g., through PD-L1/PD-L2 overexpression), aberrant expression of chemokine receptors, and alterations of the endothelial microenvironment that provide growth and survival signals [91,92]. The impaired expression of adhesion molecules contributes to intravascular luminal retention and selective tropism to endometrial tissue [93].

Factors that presumably contribute to the development of PE-PTCL include chronic endometritis, which can lead to chronic antigenic stimulation, dysregulation of T-cell receptor (TCR) signaling, and the initiation of clonal T-cell proliferation within a permissive stromal environment [21,94,95]. This can be followed by the acquisition of genetic and epigenetic aberrations that disrupt T-cell homeostasis [96]. In some PTCL subtypes, oncogenic viruses such as the Epstein–Barr virus (EBV) may also play a pathogenic role [90]. Several case reports indicate that PELs can be concealed within benign-appearing endometrial polyps [34,36,48,53,54,55,56]. Therefore, the possibility of lymphoma should be considered when endometrial polyps exhibit a dense lymphoid infiltrate. Chemotherapy or radiotherapy for endometrial carcinoma can occasionally lead to the development of secondary endometrial lymphomas [35]. Rare cases of PELs can coexist with or follow endometrial carcinomas [35,59,62]. These lymphomas may be clinically silent and can remain undetected without proper tissue analysis, underscoring the importance of continued monitoring even after the remission of carcinomas [62].

## 5. Limitations of Our Study and Future Directions

Our study has some limitations. First, the literature search was confined to a single database, PubMed. The inclusion criteria were restricted to case reports and case series published in English. Future research should remove language restrictions and incorporate additional databases, such as EMBASE, LILACS, the Cochrane Library, SCOPUS, and the Web of Science. Furthermore, a further limitation is the absence of statistical tests for bias analysis, as our narrative synthesis did not utilize meta-analytic methods. We encourage future systematic reviews that include quantitative syntheses to perform statistical assessments (e.g., funnel plots, Egger’s test) to better evaluate potential publication bias.

## 6. Conclusions

In conclusion, our study highlights several important observations. PELs typically present as a sole lesion in most cases. However, an occasional association with endometrial carcinoma can occur. AUB, vaginal spotting or irregular cycles were the most common clinical presentations. The diagnosis of PELs was established via endometrial biopsy, and immunohistochemistry was essential for diagnostic confirmation and subclassification. Immunohischemically, PE-MZL and PE-DLBCL were the most frequently reported PELs. Other exceedingly rare subtypes included PE-ENKTL, T-cell lymphoma, and PE-IVLBCL. For provisional diagnosis, staging, and follow-up, imaging is indispensable. The main modalities are US, CT, MRI, and 18F-FDG PET/CT.

## Figures and Tables

**Table 1:** Lymphoid and dendritic cell types in the normal human endometrium: relative abundance, functional roles, and cyclical changes during the menstrual cycle.

Cell Types	Markers	Locations	Functional Roles	Cyclical Changes (Numbers of Cells)	References
**CD8^+^ T** Lymphocytes	CD3^+^ CD8^+^	Endometrial stroma and epithelium	Immune surveillance and cytotoxicity	Moderate during the proliferative phase and increased during the secretory phase	[2,3,4,5]
**CD4^+^ T Helper** Lymphocytes	CD3^+^ CD4^+^	Scattered in the endometrial stroma	Immune regulation and cytokine secretion	Varies throughout the cycle, with a rise post-ovulation	[6]
**Regulatory T** Lymphocytes	FOXP3^+^, CD25^+^	Endometrial stroma	Enhancing the immune tolerance during implantation	Increases in the mid-secretory phase	[7]
**NK Cells**	CD56**^+^** CD16^−^	Perivascular endometrial stroma	Spiral artery remodeling, cytokine secretion, and immune modulation	Low in the proliferative phase, peaking in the mid-to-late secretory phase, and dropping after menstruation	[8,9,10]
**Macrophages**	CD68^+^, CD163^+^, CD14^+^	Throughout the endometrial stroma	Phagocytosis, tissue repair, cytokine production, and angiogenesis	Moderate in the early cycle with an increase in the secretory phase, peaking at menstruation for tissue breakdown and repair	[3,5,8,11]
**Dendritic Cells (DCs)**	CD11c^+^, HLA-DR^+^, CD83^+^, CD1a^+^	Endometrial basalis layer, and stroma	Antigen presentation, induction of tolerance, and interaction with T lymphocytes and NK cells	Immature DCs predominate, with mature forms increasing in the late secretory phase	[12,13]
**B** Lymphocytes	CD20^+^	Rare in healthy endometrial tissue	Humoral immunity (limited role)	Usually absent or extremely low in the normal human endometrium	[2,14,15]

**Table 2:** Primary endometrial marginal zone lymphomas.

Study No.	Type of Lymphoma	Age (Yrs)	Number of Cases	Clincal Presentation	References
**1**	PE-MZL	61	1	AUB	[68]
**2**	PE-MZL	60	1	AUB	[56]
**3**	PE-MZL	70	1	AUB	[69]
**4**	PE-MZL	62 (average age)	8	AUB	[31]
**5**	PE-MZL	49 (average age)	4	AUB	[33]
**6**	PE-MZL	-	1	AUB	[70]
**7**	PE-MALT lymphoma	81	1	AUB	[71]
**8**	PE-MZL	61	1	AUB	[72]
**9**	PE-MZL	46 (average age)	1	AUB	[73]
**10**	PE-MZL	55	1	AUB	[74]
**11**	PE-MZL	65	1	AUB	[55]
**12**	PE-MZL	55 (average age)	1	AUB	[75]

PE-MZL: Primary extranodal marginal zone lymphoma of the endometrium; PE-MALT lymphoma: Primary endometrial mucosa-associated lymphoid tissue lymphoma; AUB: Abnormal uterine bleeding.

**Table 3:** Primary endometrial diffuse large B-cell lymphomas.

Study No.	Type of Lymphoma	Age (Yrs)	Number of Cases	Clincal Presentation	References
**1**	PE-DLBCL (Endometrial polyp)	49	1	AUB	[34]
**2**	PE-PBL	29	1	AUB	[76]
**3**	PE-DLBCL	88	1	AUB	[35]
**4**	PE-B-cell lymphoma (Endometrial polyp)	53	1	AUB	[53]
**5**	PE-DLBCL (Endometrial polyp)	56	1	AUB	[77]
**6**	PE-DLBCL (Transformed from PE-MZL)	66	1	AUB	[58]
**7**	PE-DLBCL	67	1	AUB	[57]
**8**	PE-DLBCL (Endometrial polyp)	89	1	AUB	[54]
**9**	PE-DLBCL	Menopausal	1	AUB	[78]
**10**	PE-DLBCL	46 (Average age)	3	AUB	[73]
**11**	PE-DLBCL (Endometrial polyp)	44	1	AUB	[36]
**12**	Carcinoma with PELs (2 PE-DLBCL, 1 PE-FL)	56	3	AUB	[62]
**13**	PE-DLBCL	59	1	AUB	[79]
**14**	PE-Immunoblastic NHL (HIV-associated)	35	1	AUB	[80]
**15**	PE-DLBCL	31 and 36	2	AUB	[38]

PE-DLBCL: Primary endometrial diffuse large B-cell lymphoma; PE-PBL: Primary endometrial plasmablastic lymphoma; PE-FL: Primary endometrial follicular lymphoma; PE-MZL: Primary endometrial marginal zone lymphoma; HIV-associated: Human immunodeficiency virus-associated; NHL: Non-Hodgkin lymphoma; AUB: Abnormal uterine bleeding.

**Table 4:** Other types of primary endometrial lymphomas.

Study No.	Type of Lymphoma	Age (Yrs)	Number of Cases	Clincal Presentation	References
**1**	PE-IVLBCL	32	1	AUB	[81]
**2**	PE-IVDLBCL	66	1	AUB	[48]
**3**	PE-IVDLBCL	47	1	AUB	[47]
**4**	PE-IVDLBCL	62	1	AUB	[46]
**5**	PE-ENKTL	44	1	AUB	[50]
**6**	PE-ENKTL	48	1	AUB	[49]
**7**	PE-ENKTL	40	1	AUB	[82]
**8**	PE-BL	65	1	AUB	[39]
**9**	PE-BL	6	1	AUB	[40]
**10**	PE-FL	67	1	AUB	[59]
**11**	PE-low-grade NHL	62	1	AUB	[83]
**12**	PE-low-grade NHL	59	3	AUB	[84]
**13**	PE-T-ALL/LBL	38	1	AUB	[52]
**14**	PE-TCL	75	1	AUB	[51]
**15**	PE-TCL	68	1	AUB	[85]
**16**	PE-HL	30	1	AUB	[86]

PE-IVLBCL: Primary endometrial intravascular large B-cell lymphoma; PE-ENKTL: Primary endometrial extranodal natural killer/T-cell lymphoma; PE-BL: Primary endometrial Burkitt lymphoma; PE-FL: Primary endometrial follicular lymphoma; PE-low-grade NHL: Primary endometrial low-grade non-Hodgkin lymphoma; PE-T-ALL/LBL: Primary endometrial T-cell acute lymphoblastic lymphoma/leukemia; PE-TCL: Primary endometrial T-cell lymphoma; PE-HL: Primary endometrial Hodgkin lymphoma; AUB: Abnormal uterine bleeding; NHL: Non-Hodgkin lymphoma.

**Table 5:** Primary endometrial lymphoma presenting within endometrial polyps.

Study No.	Type of Lymphoma	Age (Yrs)	Number of Cases	Clincal Presentation	References
**1**	PE-DLBCL	49	1	Incidental finding	[34]
**2**	PE-B-cell lymphoma	53	1	Vaginal spotting	[53]
**3**	PE-DLBCL	56	1	Vaginal bleeding	[77]
**4**	PE-DLBCL	89	1	Vaginal bleeding	[54]
**5**	PE-DLBCL	44	1	Vaginal spotting	[36]
**6**	PE-MZL (MALT lymphoma)	60	1	Vaginal bleeding	[56]
**7**	PE-MZL (MALT lymphoma)	65	1	Vaginal bleeding	[55]
**8**	PE-IVLBCL	66	1	Vaginal bleeding	[48]

PE-IVLBCL: Primary endometrial intravascular large B-cell lymphoma; PE-DLBCL: Primary endometrial diffuse large B-cell lymphoma; PE-MZL (MALT lymphoma): Primary endometrial marginal zone lymphoma (mucosa-associated lymphoid tissue lymphoma).

## Data Availability

The original contributions presented in this study are included in the article/Supplementary Materials. Further inquiries can be directed to the corresponding author.

## Supplementary Materials

The following supporting information can be downloaded at: https://www.mdpi.com/article/10.3390/diagnostics16060849/s1, Prisma 2020 Checklist.

## Abbreviations

PE	Primary endometrial
PELs	Primary endometrial lymphomas
NK cells	Natural killer cells
DCs	Dendritic cells
ELAs	Endometrial lymphoid aggregates
uNK cells	Uterine natural killer cells
PE-DLBCL	Primary endometrial diffuse large B-cell lymphoma
PE-MZL	Primary endometrial marginal zone lymphoma
MALT lymphoma	Mucosa-associated lymphoid tissue lymphoma
PE-FL	Primary endometrial follicular lymphoma
PE-ENKTL	Primary endometrial extranodal NK/T-cell lymphoma
PE-IVLBCL	Primary endometrial intravascular large B-cell lymphoma
PE-PTCL	Primary endometrial peripheral T-cell lymphoma
NHL	Non-Hodgkin lymphoma
PE-low-grade NHL	Primary endometrial low-grade non-Hodgkin lymphoma
PE-T-ALL/LBL	Primary endometrial precursor T-cell acute lymphoblastic leukemia/lymphoma
PE-TCL	Primary endometrial T-cell lymphoma
PE-HL	Primary endometrial Hodgkin lymphoma
AUB	Abnormal uterine bleeding
US	Ultrasonography
CT	Computed tomography
MRI	Magnetic resonance imaging
FDG-PET/CT	18F-fluoro-2-deoxyglucose positron emission tomography/computed tomography
PET-CT	Positron emission tomography-computed tomography
ADC	Apparent diffusion coefficient
DWI	Diffusion-weighted imaging
