# Supplementary material for: Primary Endometrial Lymphomas: A Systematic Review
Source: Diagnostics (Basel). 2026 Mar 12;16(6):849. doi: 10.3390/diagnostics16060849 (PMC13025808; doi:10.3390/diagnostics16060849)
Supplement: Supplementary file 1 [file diagnostics-16-00849-s001.zip › diagnostics-4091851-supplementary.pdf]

PRISMA 2020 Checklist

The completed PRISMA 2020 checklist:

Tuesday, February 24, 2026

PRISMA 2020 Checklist - Completed

Review Title: Primary Endometrial Lymphomas: A Systematic Review

**Section and Topic**	**Item #**	**Checklist Item**	**Location where item is reported**
**TITLE**	1	Identify the report as a systematic review.	The report was identified as a systematic review**:**<br>Lines: 2
**ABSTRACT**	2	See the PRISMA 2020 for Abstracts checklist.	We followed PRISMA 2020. The abstract has a structured format covering Background, Objective, Methods, Results, and Conclusions.<br>Lines:19-37
**INTRODUCTION**			
Rationale	3	Describe the rationale for the review in the context of existing knowledge.	Rational for the review was described in "**Introduction section**":<br>Lines: 358-366
Objectives	4	Provide an explicit statement of the objective(s) or question(s) the review addresses.	An explicit statement of the objective(s) was provided in the **Abstract:**<br>Lines:20-21<br>Also, explicit statement of the objective (s) was

Section and Topic	Item #	Checklist Item	Location where item is reported
			provided in the " **Introduction section”:** Lines: 358-366
**METHODS**			
Eligibility criteria	5	Specify the inclusion and exclusion criteria for the review and how studies were grouped for the syntheses.	We specified the inclusion and exclusion criteria for the review in the **“Methods section”** Lines: 387-400 Also, we specified how studies were grouped for the syntheses in the **“Methods section”:** Lines: 416-425
Information sources	6	Specify all databases, registers, websites, organisations, reference lists and other sources searched or consulted to identify studies. Specify the date when each source was last searched or consulted.	We specified all databases, registers, websites, organizations, reference lists and other sources searched or consulted to identify studies in the “**Methods section”:** Lines:381-384
Search strategy	7	Present the full search strategies for all databases, registers and websites, including any filters and limits used.	We presented full search strategies for all databases, registers and websites, including any filters and limits used in the “**Methods” section:** Lines: 375-385
Selection process	8	Specify the methods used to decide whether a study met the inclusion criteria of the review, including how many reviewers screened each record and each report retrieved,	We specified the methods used to decide whether a study met the inclusion criteria of the review, including how many reviewers screened each record and each report

Section and Topic	Item #	Checklist Item	Location where item is reported
		whether they worked independently, and if applicable, details of automation tools used in the process.	retrieved, whether they worked independently, and if applicable, details of automation tools used in the process in the "**Methods" section:** Lines: 375-385
Data collection process	9	Specify the methods used to collect data from reports, including how many reviewers collected data from each report, whether they worked independently, any processes for obtaining or confirming data from study investigators, and if applicable, details of automation tools used in the process.	We specified the methods used to collect data from reports, including how many reviewers collected data from each report, whether they worked independently, any processes for obtaining or confirming data from study investigators, and if applicable, details of automation tools used in the process in the **"Methods section":** Lines:375-385
Data items	10a	List and define all outcomes for which data were sought. Specify whether all results that were compatible with each outcome domain in each study were sought...	We listed and defined all outcomes for which data were sought in the **"Results section":** Lines:375-399
	10b	List and define all other variables for which data were sought (e.g. participant and intervention characteristics, funding sources). Describe any assumptions made about any missing or unclear information.	We listed and defined all other variables for which data were sought in the **"Methods section":** Lines:375-399 Assumptions made about any missing or unclear information: Not applicable

Section and Topic	Item #	Checklist Item	Location where item is reported
Study risk of bias assessment	11	Specify the methods used to assess risk of bias in the included studies...	We specified the methods used to assess risk of bias in the included studies...in the **"Methods section".** Lines: 410-415
Effect measures	12	Specify for each outcome the effect measure(s) (e.g. risk ratio, mean difference) used in the synthesis or presentation of results.	**Not Applicable.** This is a descriptive systematic review without a meta-analysis. Results are presented as counts, percentages, and mean ages. This was presented in the **"Result section":** Lines: 416-425
Synthesis methods	13a	Describe the processes used to decide which studies were eligible for each synthesis...	**Results:** Studies are synthesized by grouping them into tables based on lymphoma type (Tables 2-5) and clinical context (e.g., polyps, carcinoma). Lines: 416-425
	13b	Describe any methods required to prepare the data for presentation or synthesis, such as handling of missing summary statistics, or data conversions.	**Not applicable.**
	13c	Describe any methods used to tabulate or visually display results of individual studies and syntheses.	In the **"Results section",** results are presented in Tables 2-5: Lines: 416-425 Also, the results were summarized in the text, In the **"Results section"**: Lines: 416-425

Section and Topic	Item #	Checklist Item	Location where item is reported
	13d	Describe any methods used to synthesize results and provide a rationale for the choice(s). If meta-analysis was performed, describe the model(s)...	We described methods used to synthesize results and provided a rationale for the choice(s) in the "**Methods section":**<br>Lines: 416-425
	13e	Describe any methods used to explore possible causes of heterogeneity among study results (e.g. subgroup analysis, meta-regression).	**Not Applicable.**
	13f	Describe any sensitivity analyses conducted to assess robustness of the synthesized results.	**CASP was used:**<br>Lines: 410-415
Reporting bias assessment	14	Describe any methods used to assess risk of bias due to missing results in a synthesis (arising from reporting biases).	We addressed" Assessment of Study Quality and Risk of Bias*" in the "***Methods section***":*<br>Lines: 410-415
Certainty assessment	15	Describe any methods used to assess certainty (or confidence) in the body of evidence for an outcome.	**CASP was used:**<br>Lines: 410-415
**RESULTS**			
Study selection	16a	Describe the results of the search and selection process, from the number of records identified in the search to the number of studies included in the review, ideally using a flow diagram.	We described the results of the search and selection process, from the number of records identified in the search to the number of studies included in the review, ideally using a flow diagram in the **"Methods section":**

Section and Topic	Item #	Checklist Item	Location where item is reported
			Lines:375-399<br>Lines:403-409 (Figure 6)
	16b	Cite studies that might appear to meet the inclusion criteria, but which were excluded, and explain why they were excluded.	Not applicable
Study characteristics	17	Cite each included study and present its characteristics.	**Tables 2-5** cited the included studies, their reference, and key characteristics (age, lymphoma type, presentation).
Risk of bias in studies	18	Present assessments of risk of bias for each included study.	**We addressed the issue of** assessments of risk of bias**.**<br>Lines: 410-415
Results of individual studies	19	For all outcomes, present, for each study: (a) summary statistics for each group (where appropriate) and (b) an effect estimate and its precision...	**Tables 2-5** cited the included studies, their reference, and key characteristics (age, lymphoma type, presentation).<br>Lines: 410-415
Results of syntheses	20a	For each synthesis, briefly summarise the characteristics and risk of bias among contributing studies.	**Results:** The text summarizes the findings from the synthesized tables (e.g., "PE-MZL was the most frequently reported subtype...") in the **"Result section"**. Risk of bias is not summarized.<br>Lines:416-425
	20b	Present results of all statistical syntheses conducted. If meta-	Descriptive statistics were performed in the **"Result section".**

Section and Topic	Item #	Checklist Item	Location where item is reported
		analysis was done, present for each the summary estimate and its precision...	Lines: 416-425
	20c	Present results of all investigations of possible causes of heterogeneity among study results.	**Not Applicable.**
	20d	Present results of all sensitivity analyses conducted to assess the robustness of the synthesized results.	We addressed this issue in the "Methods section" , CASP<br>Lines: 410-415
Reporting biases	21	Present assessments of risk of bias due to missing results (arising from reporting biases) for each synthesis assessed.	**Not applicable**
Certainty of evidence	22	Present assessments of certainty (or confidence) in the body of evidence for each outcome assessed.	We addressed this issue in the "Methods section" , CASP<br>Lines: 410-415
**DISCUSSION**			
Discussion	23a	Provide a general interpretation of the results in the context of other evidence.	We provided a general interpretation of the results in the context of other evidence in the "**Discussion section":**<br>Lines:547-587
	23b	Discuss any limitations of the evidence included in the review.	We discussed any limitations of the evidence included in the review in the **"Discussion section".**<br>Lines:580-587

Section and Topic	Item #	Checklist Item	Location where item is reported
	23c	Discuss any limitations of the review processes used.	We discussed any limitations of the evidence included in the review in the **“Discussion section”.**<br>Lines:581-588
	23d	Discuss implications of the results for practice, policy, and future research.	**Discussion:** The conclusion and the discussion of each lymphoma subtype implicitly cover implications for diagnosis and consideration in differential diagnoses.<br>Lines:180-184<br>Lines: 211-216<br>Lines: 227-231<br>Lines: 251-252<br>Lines: 270-274<br>Lines: 289-291
**OTHER INFORMATION**			
Registration and protocol	24a	Provide registration information for the review, including register name and registration number, or state that the review was not registered.	We provided registration information for the review, including register name and registration number in the **“Materials and method section”.**<br>Lines:372-374
	24b	Indicate where the review protocol can be accessed, or state that a protocol was not prepared.	We provided registration information for the review, including register name and registration number in the **“Materials and method section”.**<br>Lines: 372-374
	24c	Describe and explain any amendments to information provided at	**Not applicable.**

Section and Topic	Item #	Checklist Item	Location where item is reported
		registration or in the protocol.	
Support	25	Describe sources of financial or non-financial support for the review, and the role of the funders or sponsors in the review.	**Funding section:** "This research received no external funding" Lines:632
Competing interests	26	Declare any competing interests of review authors.	**Conflicts of Interest section:** "The authors declare no conflict of interest." Lines:636
Availability of data, code and other materials	27	Report which of the following are publicly available and where they can be found: template data collection forms; data extracted from included studies; data used for all analyses; analytic code; any other materials used in the review.	**Availability of data and material section:** "All data and materials are included inside the manuscript." Lines:635

Excluded studies

1. Suzuki, H., K. Hamada, J. Hamanishi, A. Ueda, R. Murakami, M. Taki, R. Mizuno, K. Watanabe, H. Sato, Y. Hosoe, H. Ito, K. Yamanoi, H. Yoshitomi, N. Kakiuchi, K. Yamaguchi, N. Matsumura, S. Ogawa, H. Ueno, and M. Mandai. "Artificial Intelligence-Based Spatial Analysis of Tertiary Lymphoid Structures and Clinical Significance for Endometrial Cancer." Cancer Immunol Immunother 74, no. 3 (2025): 84.
2. Segura-Rivera, R., and S. Pina-Oviedo. "Marginal Zone Lymphoma of Extranodal Sites: A Review with an Emphasis on Diagnostic Pitfalls and Differential Diagnosis with Reactive Conditions." Hum Pathol 156 (2025): 105683.
3. Nagase, Y., M. Kodama, E. Aimono, K. Nakamura, R. Takamatsu, K. Abe, T. Yoshimura, T. Chiyoda, W. Yamagami, and H. Nishihara. "Cxcl9 and Cxcl13 Shape

Endometrial Cancer Immune-Activated Microenvironment Via Tertiary Lymphoid Structure Formation." Cancer Sci (2025).
4. Niu, S., H. Lu, W. Li, and Y. Hou. "Immunohistochemical Expression of Lymphoid Enhancer-Binding Factor 1 in Low-Grade Endometrial Stromal Tumors." Int J Gynecol Pathol 43, no. 5 (2024): 487-93.
5. Medeiros, L. J., A. Chadburn, Y. Natkunam, K. N. Naresh, and W. H. O. th Edition Classification Project. "Fifth Edition of the World Health Classification of Tumors of the Hematopoietic and Lymphoid Tissues: B-Cell Neoplasms." Mod Pathol 37, no. 4 (2024): 100441.
6. Marshall, E. H., B. Brumbaugh, A. Holt, S. T. Chen, and M. P. Hoang. "Cutaneous Intravascular Hematolymphoid Entities: A Review." Diagnostics (Basel) 14, no. 7 (2024).
7. Koyama, S., H. Okamoto, K. Yamanoi, R. Mizuno, M. Sunada, M. Taki, R. Murakami, H. Ito, K. Yamaguchi, J. Hamanishi, and M. Mandai. "A Case of Grade1 Follicular Lymphoma Diagnosed by Laparoscopic Lymph Node Resection: Differentiating from Late Lymph Node Recurrence of Endometrial Cancer." Int Cancer Conf J 13, no. 4 (2024): 525-31.
8. Jia, H. Q., S. P. Zhang, Y. Chen, Y. H. Qiao, Y. F. Yao, X. Y. Zhang, S. Y. Wu, Y. L. Song, and X. M. Xing. "Characteristics and Significance of Tertiary Lymphoid Structures Based on Molecular Subtypes in Endometrial Cancer." Int J Gynecol Pathol 43, no. 6 (2024): 595-604.
9. Ghorbanniadelavar, Z., M. Jalali Nadoushan, and M. Soltanipur. "Comparison of B-Cell Lymphoma 2 (Bcl-2) Expression in Disordered Proliferative Endometrium and Simple Endometrial Hyperplasia." Rev Esp Patol 57, no. 4 (2024): 265-72.
10. Yokoi, A., Y. Nakamura, M. Hashimura, Y. Oguri, T. Matsumoto, M. Nakagawa, Y. Ishibashi, T. Ito, K. Ohhigata, Y. Harada, N. Fukagawa, and M. Saegusa. "Anaplastic Lymphoma Kinase Overexpression Enhances Aggressive Phenotypic Characteristics of Endometrial Carcinoma." BMC Cancer 23, no. 1 (2023): 765.
11. Huang, D., M. Chan, M. Solomon, M. I. Cedars, L. C. Giudice, and H. Cakmak. "B-Cell Lymphoma 6 Expression Significantly Differs by the Uterine Preparation Method Used for Frozen Embryo Transfer." Fertil Steril 120, no. 2 (2023): 305-11.
12. Gonzalez-Farre, B., J. E. Ramis-Zaldivar, N. Castrejon de Anta, A. Rivas-Delgado, F. Nadeu, J. Salmeron-Villalobos, A. Enjuanes, K. Karube, O. Balague, F. Cobo, N. Kelleher, I. Victoria, L. Veloza, C. Teixido, E. Gine, M. Lopez-Guerra, L. Quintanilla-Martinez, A. Lopez-Guillermo, I. Salaverria, and E. Campo. "Intravascular Large B-Cell Lymphoma Genomic Profile Is Characterized by Alterations in Genes Regulating Nf-Kappab and Immune Checkpoints." Am J Surg Pathol 47, no. 2 (2023): 202-11.
13. Sugahara, T., Y. Tanaka, M. Hamaguchi, M. Fujii, K. Shimura, K. Ogawa, T. Mori, I. Kusuki, M. Fukui, and J. Kitawaki. "Reduced Innate Lymphoid Cells in the Endometrium of Women with Endometriosis." Am J Reprod Immunol 87, no. 1 (2022): e13502.
14. Shen, M., E. O'Donnell, G. Leon, A. Kisovar, P. Melo, K. Zondervan, I. Granne, and J. Southcombe. "The Role of Endometrial B Cells in Normal Endometrium and Benign Female Reproductive Pathologies: A Systematic Review." Hum Reprod Open 2022, no. 1 (2022): hoab043.
15. Rempuia, V., B. Anima, M. Jeremy, G. Gurusubramanian, P. P. Pankaj, R. K. Kharwar, and V. K. Roy. "Effects of Metformin on the Uterus of D-Galactose-Induced Aging

Mice: Histomorphometric, Immunohistochemical Localization (B-Cell Lymphoma 2, Bcl2-Associated X Protein, and Active Capase3), and Oxidative Stress Study." J Exp Zool A Ecol Integr Physiol 337, no. 6 (2022): 600-11.
16. Qin, M., J. Hamanishi, M. Ukita, K. Yamanoi, S. Takamatsu, K. Abiko, R. Murakami, T. Miyamoto, H. Suzuki, A. Ueda, Y. Hosoe, A. Horie, K. Yamaguchi, and M. Mandai. "Tertiary Lymphoid Structures Are Associated with Favorable Survival Outcomes in Patients with Endometrial Cancer." Cancer Immunol Immunother 71, no. 6 (2022): 1431-42.
17. Klimczak, A. M., N. S. Herlihy, C. S. Scott, B. M. Hanson, J. G. Kim, S. Titus, E. Seli, and R. T. Scott, Jr. "B-Cell Lymphoma 6 Expression Is Not Associated with Live Birth in a Normal Responder in Vitro Fertilization Population." Fertil Steril 117, no. 2 (2022): 351-58.
18. Kim, H., and Y. H. Ko. "The Pathologic and Genetic Characteristics of Extranodal Nk/T-Cell Lymphoma." Life (Basel) 12, no. 1 (2022).
19. Horeweg, N., H. H. Workel, D. Loiero, D. N. Church, L. Vermij, A. Leon-Castillo, R. T. Krog, S. M. de Boer, R. A. Nout, M. E. Powell, L. R. Mileshkin, H. MacKay, A. Leary, N. Singh, I. M. Jurgenliemk-Schulz, Vthbm Smit, C. L. Creutzberg, V. H. Koelzer, H. W. Nijman, T. Bosse, M. de Bruyn, and Portec consortium Trans. "Tertiary Lymphoid Structures Critical for Prognosis in Endometrial Cancer Patients." Nat Commun 13, no. 1 (2022): 1373.
20. Clark, D. A., A. Reihani, J. L. Arredondo, K. Ask, and W. G. Foster. "Cd200s-Positive Granulated Lymphoid Cells in Endometrium Appear to Be Cd56-Positive Uterine Nk Cells." J Reprod Immunol 150 (2022): 103477.
21. Chen, B., T. Mao, X. Qin, W. Zhang, N. Watanabe, and J. Li. "Role of Estrogen Receptor Signaling Pathway-Related Genes in Diffuse Large B-Cell Lymphoma and Identification of Key Targets Via Integrated Bioinformatics Analysis and Experimental Validation." Front Oncol 12 (2022): 1029998.
22. Sweeney, K. J., M. T. Tetzlaff, F. Vega, A. Gillenwater, Z. Zuo, N. Gross, P. Nagarajan, J. Wargo, K. Nelson, V. G. Prieto, C. A. Torres-Cabala, and J. L. Curry. "Tertiary Lymphoid Structures with Overlapping Histopathologic Features of Cutaneous Marginal Zone Lymphoma During Neoadjuvant Cemiplimab Therapy Are Associated with Antitumor Response." J Cutan Pathol 48, no. 5 (2021): 674-79.
23. Shimada, K., and H. Kiyoi. "Current Progress and Future Perspectives of Research on Intravascular Large B-Cell Lymphoma." Cancer Sci 112, no. 10 (2021): 3953-61.
24. Patriarca, A., and G. Gaidano. "Intravascular Lymphoma: From Vessels to Genes." Blood 137, no. 11 (2021): 1438-39.
25. Marinkovic, T., and D. Marinkovic. "Biological Mechanisms of Ectopic Lymphoid Structure Formation and Their Pathophysiological Significance." Int Rev Immunol 40, no. 4 (2021): 255-67.
26. Huhn, O., X. Zhao, L. Esposito, A. Moffett, F. Colucci, and A. M. Sharkey. "How Do Uterine Natural Killer and Innate Lymphoid Cells Contribute to Successful Pregnancy?" Front Immunol 12 (2021): 607669.
27. Ensor, A. M., C. G. Sanchez, J. E. Ensor, and K. Anand. "Primary Diffuse Large B-Cell Lymphoma of the Uterus: A Seer Database Analysis." Medicine (Baltimore) 100, no. 40 (2021): e27359.

28. Du, E., X. Qu, W. Xu, and H. Lu. "Primary Female Genital System Lymphoma Complicated by a Recurrent Mucinous Borderline Ovarian Tumor: A Case Report and Review of the Literature." BMC Womens Health 21, no. 1 (2021): 420.
29. Zhang, Y., D. Lee, T. Brimer, M. Hussaini, and L. Sokol. "Genomics of Peripheral T-Cell Lymphoma and Its Implications for Personalized Medicine." Front Oncol 10 (2020): 898.
30. Xu, H., X. Su, Y. Zhao, L. Tang, J. Chen, and G. Zhong. "Innate Lymphoid Cells Are Required for Endometrial Resistance to Chlamydia Trachomatis Infection." Infect Immun 88, no. 7 (2020).
31. Wang, Z. Z., J. Song, H. Wang, J. X. Li, Q. Xiao, Z. Yu, J. X. Liu, and Z. Liu. "B Cell-Activating Factor Promotes B Cell Survival in Ectopic Lymphoid Tissues in Nasal Polyps." Front Immunol 11 (2020): 625630.
32. Fratoni, S., M. Zanelli, M. Zizzo, F. Sanguedolce, V. Aimola, G. Cerrone, L. Ricci, A. Filosa, G. Martino, A. M. Fara, V. Annessi, A. Soriano, and S. Ascani. "The Broad Landscape of Follicular Lymphoma: Part Ii." Pathologica 112, no. 2 (2020): 79-92.
33. Fratoni, S., M. Zanelli, M. Zizzo, F. Sanguedolce, V. Aimola, G. Cerrone, L. Ricci, A. Filosa, G. Martino, and S. Ascani. "The Broad Landscape of Follicular Lymphoma: Part I." Pathologica 112, no. 1 (2020): 1-16.
34. Sugimoto, M., K. Koyama, T. Ichimura, T. Shimono, Y. Hashiguchi, and Y. Miki. "Comparison of Mr Imaging Features of Uterine Neuroendocrine Carcinoma and Uterine Malignant Lymphoma." Abdom Radiol (NY) 44, no. 10 (2019): 3377-87.
35. Ma, J., J. Yang, S. Lv, M. Gao, Y. Sun, Z. J. Chen, and C. Zhang. "Dysfunction of B-Cell Lymphoma 2/Adenovirus E1b 19kd Interacting Protein 3 in Decidua Is Involved in the Pathogenesis of Preeclampsia." J Hypertens 37, no. 10 (2019): 2048-60.
36. Huang, Y., S. Yan, L. Liu, J. Lu, X. Yang, Y. He, and J. Yun. "Incidental Lymphoma in Lymph Node Dissection for Carcinoma in the Abdominopelvic Cavity: A Single-Institution Experience." Virchows Arch 475, no. 3 (2019): 365-72.
37. Harabuchi, Y., M. Takahara, K. Kishibe, T. Nagato, and T. Kumai. "Extranodal Natural Killer/T-Cell Lymphoma, Nasal Type: Basic Science and Clinical Progress." Front Pediatr 7 (2019): 141.
38. Bulmer, J. N., and G. E. Lash. "Uterine Natural Killer Cells: Time for a Re-Appraisal?" F1000Res 8 (2019).
39. Yoon, S. N. "Follicular Lymphoma Mimicking Metastatic Nodes on the F-18 Fdg Pet/Ct and Mri for Staging of Endometrial Cancer." Nucl Med Mol Imaging 52, no. 2 (2018): 159-61.
40. Wakahashi, S., F. Kawakami, K. Wakahashi, K. Minagawa, K. Matsuo, Y. Katayama, H. Yamada, T. Matsui, and T. Sudo. "Transformed Follicular Lymphoma (Tfl) Predicts Outcome in Advanced Endometrial Cancer." Cancer Epidemiol Biomarkers Prev 27, no. 8 (2018): 963-69.
41. Travaglino, A., A. Raffone, G. Saccone, L. Insabato, A. Mollo, G. De Placido, and F. Zullo. "Loss of B-Cell Lymphoma 2 Immunohistochemical Expression in Endometrial Hyperplasia: A Specific Marker of Precancer and Novel Indication for Treatment: A Systematic Review and Meta-Analysis." Acta Obstet Gynecol Scand 97, no. 12 (2018): 1415-26.
42. Ponzoni, M., E. Campo, and S. Nakamura. "Intravascular Large B-Cell Lymphoma: A Chameleon with Multiple Faces and Many Masks." Blood 132, no. 15 (2018): 1561-67.

43. Miura, L. Y., M. A. D. Maure, M. T. Zomer, R. Ribeiro, T. C. S. Cavalcanti, and W. Kondo. "A Case of Primary Uterina Lymphoma Presenting with Bleeding, Pelvic Pain, and Dysmenorrhea." Case Rep Obstet Gynecol 2018 (2018): 5065738.
44. Lone, W., A. Alkhiniji, J. Manikkam Umakanthan, and J. Iqbal. "Molecular Insights into Pathogenesis of Peripheral T Cell Lymphoma: A Review." Curr Hematol Malig Rep 13, no. 4 (2018): 318-28.
45. Ekanayake, C. D., R. Punchihewa, and P. S. Wijesinghe. "An Atypical Presentation of an Ovarian Lymphoma: A Case Report." J Med Case Rep 12, no. 1 (2018): 338.
46. Dluski, D., D. Lewkowicz, B. Leszczynska-Gorzelak, B. Obrzut, T. Rechberger, and A. Semczuk. "An Unusual Coexistence of Chronic Lymphocytic Leukemia/Small Lymphocytic Lymphoma with Endometrioid-Type Endometrial Cancer in a 58-Year-Old Woman: A Case Study with Literature Review." Case Rep Oncol 11, no. 2 (2018): 347-52.
47. Banas, T., K. Pitynski, K. Okon, and A. Winiarska. "Non-Endometrioid and High-Grade Endometrioid Endometrial Cancers Show DNA Fragmentation Factor 40 (Dff40) and B-Cell Lymphoma 2 Protein (Bcl2) Underexpression, Which Predicts Disease-Free and Overall Survival, but Not DNA Fragmentation Factor 45 (Dff45) Underexpression." BMC Cancer 18, no. 1 (2018): 418.
48. Banas, T., K. Pitynski, K. Okon, M. Mikos, A. I. Czerw, A. Deptala, and A. Ludwin. "Immunoexpression of DNA Fragmentation Factor 40, DNA Fragmentation Factor 45, and B-Cell Lymphoma 2 Protein in Normal Human Endometrium and Uterine Myometrium Depends on Menstrual Cycle Phase and Menopausal Status." Arch Med Sci 14, no. 6 (2018): 1254-62.
49. Banas, T., K. Pitynski, M. Mikos, and J. Cielecka-Kuszyk. "Endometrial Polyps and Benign Endometrial Hyperplasia Have Increased Prevalence of DNA Fragmentation Factors 40 and 45 (Dff40 and Dff45) Together with the Antiapoptotic B-Cell Lymphoma (Bcl-2) Protein Compared with Normal Human Endometria." Int J Gynecol Pathol 37, no. 5 (2018): 431-40.
50. Onaindia, A., L. J. Medeiros, and K. P. Patel. "Clinical Utility of Recently Identified Diagnostic, Prognostic, and Predictive Molecular Biomarkers in Mature B-Cell Neoplasms." Mod Pathol 30, no. 10 (2017): 1338-66.
51. Ladikou, E. E., and E. Kassi. "The Emerging Role of Estrogen in B Cell Malignancies." Leuk Lymphoma 58, no. 3 (2017): 528-39.
52. Hatayama, Y., M. Aoki, H. Kawaguchi, K. Hirose, M. Sato, H. Akimoto, M. Tanaka, I. Fujioka, S. Ono, and Y. Takai. "Safe and Successful Birth Following Pelvic Radiotherapy for Rectal Mucosa-Associated Lymphoid Tissue Lymphoma: A Case Report." J Med Case Rep 11, no. 1 (2017): 26.
53. Salamonsen, L. A., J. Evans, H. P. Nguyen, and T. A. Edgell. "The Microenvironment of Human Implantation: Determinant of Reproductive Success." Am J Reprod Immunol 75, no. 3 (2016): 218-25.
54. Lax, S. F. "[Endometritis : Rare Disease with Clinical Importance?]." Pathologe 37, no. 6 (2016): 521-25.
55. Ainsworth, A., A. Wood, P. Kurtin, and T. Burnett. "An Unusual Case of Abnormal Uterine Bleeding Due to Classical Hodgkin Lymphoma Identified by Endometrial Biopsy." Int J Gynaecol Obstet 135, no. 3 (2016): 331.

56. Okuda, T., S. Ijichil, S. Yamashita, T. Yoshioka, H. Nishigaki, and J. Kitawaki. "Diagnostic Usefulness of Fdg-Pet/Ct in Advanced Malignant Lymphoma of the Uterus: Report of Two Cases." Eur J Gynaecol Oncol 36, no. 6 (2015): 737-41.
57. Doisne, J. M., E. Balmas, S. Boulenouar, L. M. Gaynor, J. Kieckbusch, L. Gardner, D. A. Hawkes, C. F. Barbara, A. M. Sharkey, H. J. Brady, J. J. Brosens, A. Moffett, and F. Colucci. "Composition, Development, and Function of Uterine Innate Lymphoid Cells." J Immunol 195, no. 8 (2015): 3937-45.
58. Jasper, M. J., K. P. Tremellen, and S. A. Robertson. "Primary Unexplained Infertility Is Associated with Reduced Expression of the T-Regulatory Cell Transcription Factor Foxp3 in Endometrial Tissue." Mol Hum Reprod 12, no. 5 (2006): 301-8.
59. Zarnani, A. H., S. M. Moazzeni, F. Shokri, M. Salehnia, and M. Jeddi Tehrani. "Analysis of Endometrial Myeloid and Lymphoid Dendritic Cells During Mouse Estrous Cycle." J Reprod Immunol 71, no. 1 (2006): 28-40.
60. Lagoo, A. S., and S. J. Robboy. "Lymphoma of the Female Genital Tract: Current Status." Int J Gynecol Pathol 25, no. 1 (2006): 1-21.
61. Pals, S. T., D. J. de Gorter, and M. Spaargaren. "Lymphoma Dissemination: The Other Face of Lymphocyte Homing." Blood 110, no. 9 (2007): 3102-11.
62. Jiang, X. F., K. X. Yang, Z. L. Peng, L. Xu, Q. Huang, and Q. Li. "[Clinicopathologic and Immunohistochemical Study of Primary Non-Hodgkin Lymphoma of the Female Genital System]." Zhonghua Fu Chan Ke Za Zhi 42, no. 4 (2007): 222-6.
63. Goto, N., Y. Oishi-Tanaka, H. Tsunoda, H. Yoshikawa, and M. Minami. "Magnetic Resonance Findings of Primary Uterine Malignant Lymphoma." Magn Reson Med Sci 6, no. 1 (2007): 7-13.
64. Sentman, C. L., C. R. Wira, and M. Eriksson. "Nk Cell Function in the Human Female Reproductive Tract." Am J Reprod Immunol 57, no. 2 (2007): 108-15.
65. Geyer, J. T., J. A. Ferry, N. L. Harris, R. H. Young, J. A. Longtine, and L. R. Zukerberg. "Florid Reactive Lymphoid Hyperplasia of the Lower Female Genital Tract (Lymphoma-Like Lesion): A Benign Condition That Frequently Harbors Clonal Immunoglobulin Heavy Chain Gene Rearrangements." Am J Surg Pathol 34, no. 2 (2010): 161-8.
66. Eck, M., and W. Fischbach. "[Gastric Malt-Type Lymphoma. Pathology, Pathogenesis, Diagnostics and Therapy]." Pathologe 31, no. 3 (2010): 188-94.
67. Alduaij, A., K. Hansen, and C. Zhang. "Primary Follicular Lymphoma of the Fallopian Tube Found Incidentally in a Patient Treated for Endometrial Carcinoma: A Case Report." Diagn Pathol 5 (2010): 44.
68. Schulke, L., M. Berbic, F. Manconi, N. Tokushige, R. Markham, and I. S. Fraser. "Dendritic Cell Populations in the Eutopic and Ectopic Endometrium of Women with Endometriosis." Hum Reprod 24, no. 7 (2009): 1695-703.
69. Au, W. Y., D. D. Weisenburger, T. Intragumtornchai, S. Nakamura, W. S. Kim, I. Sng, J. Vose, J. O. Armitage, R. Liang, and T. Cell Lymphoma Project International Peripheral. "Clinical Differences between Nasal and Extranasal Natural Killer/T-Cell Lymphoma: A Study of 136 Cases from the International Peripheral T-Cell Lymphoma Project." Blood 113, no. 17 (2009): 3931-7.
70. Schulke, L., F. Manconi, R. Markham, and I. S. Fraser. "Endometrial Dendritic Cell Populations During the Normal Menstrual Cycle." Hum Reprod 23, no. 7 (2008): 1574-80.

71. Plaks, V., T. Birnberg, T. Berkutzki, S. Sela, A. BenYashar, V. Kalchenko, G. Mor, E. Keshet, N. Dekel, M. Neeman, and S. Jung. "Uterine Dcs Are Crucial for Decidua Formation During Embryo Implantation in Mice." J Clin Invest 118, no. 12 (2008): 3954-65.
72. Ma, H. L., T. Zhang, J. Meng, Z. Y. Qin, F. Du, Q. Y. Wang, and S. L. Wei. "The Role of T-Lymphoma Invasion and Metastasis Inducing Protein 1 in Early Pregnancy in Mice." Mol Hum Reprod 14, no. 10 (2008): 589-94.
73. Gaillot, L., F. Allias, G. Dubernard, F. Berger, and M. Devouassoux-Shisheboran. "[Lymphoma-Like Lesions of the Endometrium]." Ann Pathol 28, no. 6 (2008): 504-7.
74. Estrada-Sanchez, G., F. J. Ochoa-Carrillo, and J. Altamirano-Ley. "[(18)Fdg Pet/Ct Imaging in Primary Breast Lymphoma and Breast Cancer]." Cir Cir 76, no. 4 (2008): 279-86.
75. Russell, P., G. Sacks, K. Tremellen, and A. Gee. "The Distribution of Immune Cells and Macrophages in the Endometrium of Women with Recurrent Reproductive Failure. Iii: Further Observations and Reference Ranges." Pathology 45, no. 4 (2013): 393-401.
76. Hussein, M. R. "Atypical Lymphoid Proliferations: The Pathologist's Viewpoint." Expert Rev Hematol 6, no. 2 (2013): 139-53.
77. Yalta, T., E. Tastekin, F. O. Puyan, U. Usta, M. Azatcam, and S. Altaner. "Non-Hodgkin's Lymphoma: A Rare Diagnosis on Cervicovaginal Cytology." J Cytol 29, no. 2 (2012): 142-3.
78. Shetty, S., T. Bruns, C. J. Weston, Z. Stamataki, Y. H. Oo, H. M. Long, G. M. Reynolds, G. Pratt, P. Moss, S. Jalkanen, S. G. Hubscher, P. F. Lalor, and D. H. Adams. "Recruitment Mechanisms of Primary and Malignant B Cells to the Human Liver." Hepatology 56, no. 4 (2012): 1521-31.
79. Rouette, A., S. Parent, J. Girouard, V. Leblanc, and E. Asselin. "Cisplatin Increases B-Cell-Lymphoma-2 Expression Via Activation of Protein Kinase C and Akt2 in Endometrial Cancer Cells." Int J Cancer 130, no. 8 (2012): 1755-67.
80. Orwat, D. E., and N. I. Batalis. "Intravascular Large B-Cell Lymphoma." Arch Pathol Lab Med 136, no. 3 (2012): 333-8.
81. McMenamin, M., T. Lysakova-Devine, M. Wingfield, C. O'Herlihy, and C. O'Farrelly. "Endometrial Aspiration Biopsy: A Non-Invasive Method of Obtaining Functional Lymphoid Progenitor Cells and Mature Natural Killer Cells." Reprod Biomed Online 25, no. 3 (2012): 322-8.
82. Kuper-Hommel, M. J., and J. H. van Krieken. "Molecular Pathogenesis and Histologic and Clinical Features of Extranodal Marginal Zone Lymphomas of Mucosa-Associated Lymphoid Tissue Type." Leuk Lymphoma 53, no. 6 (2012): 1032-45.
83. Evans, J., and L. A. Salamonsen. "Inflammation, Leukocytes and Menstruation." Rev Endocr Metab Disord 13, no. 4 (2012): 277-88.
84. Braggio, E., A. Dogan, J. J. Keats, W. J. Chng, G. Huang, J. M. Matthews, M. J. Maurer, M. E. Law, D. S. Bosler, M. Barrett, I. S. Lossos, T. E. Witzig, and R. Fonseca. "Genomic Analysis of Marginal Zone and Lymphoplasmacytic Lymphomas Identified Common and Disease-Specific Abnormalities." Mod Pathol 25, no. 5 (2012): 651-60.
85. Russell, P., L. Anderson, D. Lieberman, K. Tremellen, H. Yilmaz, B. Cheerala, and G. Sacks. "The Distribution of Immune Cells and Macrophages in the Endometrium of Women with Recurrent Reproductive Failure I: Techniques." J Reprod Immunol 91, no. 1-2 (2011): 90-102.

86. Lee, H. B., J. C. Park, Y. S. Lee, I. C. Jeung, and E. K. Park. "Unexpected Synchronous Follicular Lymphoma of Paraaortic and Pelvic Lymph Nodes in a Patient with Endometrial Carcinoma: A Case Report." Eur J Gynaecol Oncol 32, no. 3 (2011): 334-5.
87. Male, V., T. Hughes, S. McClory, F. Colucci, M. A. Caligiuri, and A. Moffett. "Immature Nk Cells, Capable of Producing Il-22, Are Present in Human Uterine Mucosa." J Immunol 185, no. 7 (2010): 3913-8.
88. Radford, J. A., B. A. Lieberman, D. R. Brison, A. R. Smith, J. D. Critchlow, S. A. Russell, A. J. Watson, J. A. Clayton, M. Harris, R. G. Gosden, and S. M. Shalet. "Orthotopic Reimplantation of Cryopreserved Ovarian Cortical Strips after High-Dose Chemotherapy for Hodgkin's Lymphoma." Lancet 357, no. 9263 (2001): 1172-5.
89. Suzuki, Y., Y. Tamaki, M. Hasegawa, K. Maebayashi, and N. Mitsuhashi. "Magnetic Resonance Images of Primary Malignant Lymphoma of the Uterine Body: A Case Report." Jpn J Clin Oncol 30, no. 11 (2000): 519-21.
90. Salamonsen, L. A., and L. J. Lathbury. "Endometrial Leukocytes and Menstruation." Hum Reprod Update 6, no. 1 (2000): 16-27.
91. Kostopoulos, I. S., S. B. Barbanis, V. D. Kaloutsi, and C. S. Papadimitriou. "Synchronous Occurrence of Multiple Malignant Neoplasms in the Uterus (Adenocarcinoma of the Endometrium, Large B-Cell Lymphoma of the Cervix)." Pathol Res Pract 196, no. 8 (2000): 573-5.
92. Searle, R. F., R. K. Jones, and J. N. Bulmer. "Phenotypic Analysis and Proliferative Responses of Human Endometrial Granulated Lymphocytes During the Menstrual Cycle." Biol Reprod 60, no. 4 (1999): 871-8.
93. Masunaga, A., M. Abe, E. Tsujii, Y. Suzuki, T. Ohgida, M. Toyama, H. Nakamura, S. Mori, I. Sugawara, and S. Itoyama. "Primary Uterine T-Cell Lymphoma." Int J Gynecol Pathol 17, no. 4 (1998): 376-9.
94. Jones, R. K., J. N. Bulmer, and R. F. Searle. "Phenotypic and Functional Studies of Leukocytes in Human Endometrium and Endometriosis." Hum Reprod Update 4, no. 5 (1998): 702-9.
95. Yeaman, G. R., P. M. Guyre, M. W. Fanger, J. E. Collins, H. D. White, W. Rathbun, K. A. Orndorff, J. Gonzalez, J. E. Stern, and C. R. Wira. "Unique Cd8+ T Cell-Rich Lymphoid Aggregates in Human Uterine Endometrium." J Leukoc Biol 61, no. 4 (1997): 427-35.
96. White, H. D., K. M. Crassi, A. L. Givan, J. E. Stern, J. L. Gonzalez, V. A. Memoli, W. R. Green, and C. R. Wira. "Cd3+ Cd8+ Ctl Activity within the Human Female Reproductive Tract: Influence of Stage of the Menstrual Cycle and Menopause." J Immunol 158, no. 6 (1997): 3017-27.
97. Pals, S. T., P. Drillenburg, T. Radaszkiewicz, and E. Manten-Horst. "Adhesion Molecules in the Dissemination of Non-Hodgkin's Lymphomas." Acta Haematol 97, no. 1-2 (1997): 73-80.
98. Hangaishi, A., S. Ogawa, K. Mitani, N. Hosoya, S. Chiba, Y. Yazaki, and H. Hirai. "Mutations and Loss of Expression of a Mismatch Repair Gene, Hmlh1, in Leukemia and Lymphoma Cell Lines." Blood 89, no. 5 (1997): 1740-7.
99. Givan, A. L., H. D. White, J. E. Stern, E. Colby, E. J. Gosselin, P. M. Guyre, and C. R. Wira. "Flow Cytometric Analysis of Leukocytes in the Human Female Reproductive Tract: Comparison of Fallopian Tube, Uterus, Cervix, and Vagina." Am J Reprod Immunol 38, no. 5 (1997): 350-9.

100. Graf, G., U. Dehmel, and H. G. Drexler. "Expression of Thrombopoietin and Thrombopoietin Receptor Mpl in Human Leukemia-Lymphoma and Solid Tumor Cell Lines." Leuk Res 20, no. 10 (1996): 831-8.
101. Stroh, E. L., P. C. Besa, J. D. Cox, L. M. Fuller, and F. F. Cabanillas. "Treatment of Patients with Lymphomas of the Uterus or Cervix with Combination Chemotherapy and Radiation Therapy." Cancer 75, no. 9 (1995): 2392-9.
102. Rosenberg, L., J. R. Palmer, A. G. Zauber, M. E. Warshauer, B. L. Strom, S. Harlap, and S. Shapiro. "Relation of Benzodiazepine Use to the Risk of Selected Cancers: Breast, Large Bowel, Malignant Melanoma, Lung, Endometrium, Ovary, Non-Hodgkin's Lymphoma, Testis, Hodgkin's Disease, Thyroid, and Liver." Am J Epidemiol 141, no. 12 (1995): 1153-60.
103. Kawakami, S., K. Togashi, N. Kojima, K. Morikawa, T. Mori, and J. Konishi. "Mr Appearance of Malignant Lymphoma of the Uterus." J Comput Assist Tomogr 19, no. 2 (1995): 238-42.
104. Doren, M., H. P. Schneider, and W. Holzgreve. "Sonographic Manifestation of Non-Hodgkin's Lymphoma Invading the Postmenopausal Endometrium." Ultrasound Obstet Gynecol 6, no. 4 (1995): 300-1.
105. Chen, C. K., S. C. Huang, C. L. Chen, M. R. Yen, H. C. Hsu, and H. N. Ho. "Increased Expressions of Cd69 and Hla-Dr but Not of Cd25 or Cd71 on Endometrial T Lymphocytes of Nonpregnant Women." Hum Immunol 42, no. 3 (1995): 227-32.
106. Bai, P., and J. Sun. "[Primary Malignant Lymphoma of the Female Genital Tract Clinical Analysis of 15 Cases]." Zhonghua Fu Chan Ke Za Zhi 30, no. 10 (1995): 614-7.
107. Klentzeris, L. D., J. N. Bulmer, M. A. Warren, L. Morrison, T. C. Li, and I. D. Cooke. "Lymphoid Tissue in the Endometrium of Women with Unexplained Infertility: Morphometric and Immunohistochemical Aspects." Hum Reprod 9, no. 4 (1994): 646-52.
108. Johnstone, F. D., A. R. Williams, G. A. Bird, and S. Bjornsson. "Immunohistochemical Characterization of Endometrial Lymphoid Cell Populations in Women Infected with Human Immunodeficiency Virus." Obstet Gynecol 83, no. 4 (1994): 586-93.
109. Roos, E. "Adhesion Molecules in Lymphoma Metastasis." Semin Cancer Biol 4, no. 5 (1993): 285-92.
110. Monterroso, V., E. S. Jaffe, M. J. Merino, and L. J. Medeiros. "Malignant Lymphomas Involving the Ovary. A Clinicopathologic Analysis of 39 Cases." Am J Surg Pathol 17, no. 2 (1993): 154-70.
111. Aozasa, K., K. Saeki, M. Ohsawa, K. Horiuchi, K. Mishima, and M. Tsujimoto. "Malignant Lymphoma of the Uterus. Report of Seven Cases with Immunohistochemical Study." Cancer 72, no. 6 (1993): 1959-64.
112. Klentzeris, L. D., J. N. Bulmer, A. Warren, L. Morrison, T. C. Li, and I. D. Cooke. "Endometrial Lymphoid Tissue in the Timed Endometrial Biopsy: Morphometric and Immunohistochemical Aspects." Am J Obstet Gynecol 167, no. 3 (1992): 667-74.
113. Skensved, H., A. Hansen, and M. Vetner. "Immunoreactive Endometritis." Br J Obstet Gynaecol 98, no. 6 (1991): 578-82.
114. Pace, D., M. Longfellow, and J. N. Bulmer. "Characterization of Intraepithelial Lymphocytes in Human Endometrium." J Reprod Fertil 91, no. 1 (1991): 165-74.

115. Kashimura, M., S. Kusano, K. Hamasaki, and Y. Sato. "Primary Malignant Lymphoma of the Endometrium: Report of a Case and Review of the Literatures." Nihon Sanka Fujinka Gakkai Zasshi 43, no. 12 (1991): 1731-4.
116. Tabibzadeh, S. "Proliferative Activity of Lymphoid Cells in Human Endometrium Throughout the Menstrual Cycle." J Clin Endocrinol Metab 70, no. 2 (1990): 437-43.
117. Otsuki, Y., Y. Maeda, S. Magari, H. Kubo, and O. Sugimoto. "Lymphatics, Intraepithelial Lymphocytes and Endometrial Lymphoid Tissues in the Rabbit Uterus: An Electron Microscopic and Immunohistological Study." Lymphology 23, no. 3 (1990): 124-34.
118. Liang, R., E. Chiu, and S. L. Loke. "Non-Hodgkin's Lymphomas Involving the Female Genital Tract." Hematol Oncol 8, no. 5 (1990): 295-9.
119. Artiukh, E. V., A. G. Iakhnitsa, and V. I. Reshetilov. "[Lymphoid Structures in the Female Endometrium in Ontogenesis]." Arkh Anat Gistol Embriol 99, no. 12 (1990): 48-53.
120. Tabibzadeh, S. S., and P. G. Satyaswaroop. "Sex Steroid Receptors in Lymphoid Cells of Human Endometrium." Am J Clin Pathol 91, no. 6 (1989): 656-63.
121. Gilks, C. B., B. D. Acker, and P. B. Clement. "Recurrent Endometrial Adenocarcinoma: Presentation as a Splenic Mass Mimicking Malignant Lymphoma." Gynecol Oncol 33, no. 2 (1989): 209-11.
122. Marshall, R. J., and D. B. Jones. "An Immunohistochemical Study of Lymphoid Tissue in Human Endometrium." Int J Gynecol Pathol 7, no. 3 (1988): 225-35.
123. Maeda, T., H. Kamegai, and H. Mori. "Malignant Lymphoma Presenting as Initial Symptom in the Uterus. Case Report." Br J Obstet Gynaecol 95, no. 11 (1988): 1195-7.
124. Hafiz, M. A., M. Rupp, E. Khalluf, and C. Wood. "Fine Needle Aspiration Biopsy in the Evaluation of Lymphoma Presenting as an Ovarian and Uterine Mass." Acta Cytol 32, no. 4 (1988): 540-2.
125. Bulmer, J. N., D. P. Lunny, and S. V. Hagin. "Immunohistochemical Characterization of Stromal Leucocytes in Nonpregnant Human Endometrium." Am J Reprod Immunol Microbiol 17, no. 3 (1988): 83-90.
126. Young, R. H., N. L. Harris, and R. E. Scully. "Lymphoma-Like Lesions of the Lower Female Genital Tract: A Report of 16 Cases." Int J Gynecol Pathol 4, no. 4 (1985): 289-99.
127. Morris, H., J. Edwards, A. Tiltman, and M. Emms. "Endometrial Lymphoid Tissue: An Immunohistological Study." J Clin Pathol 38, no. 6 (1985): 644-52.
128. Levison, D. A. "Lymphoid Tissue and Cyclical Endometrium." J Clin Pathol 38, no. 11 (1985): 1316-7.
129. Harris, N. L., and R. E. Scully. "Malignant Lymphoma and Granulocytic Sarcoma of the Uterus and Vagina. A Clinicopathologic Analysis of 27 Cases." Cancer 53, no. 11 (1984): 2530-45.
130. Chorlton, I., R. F. Karnei, Jr., F. M. King, and H. J. Norris. "Primary Malignant Reticuloendothelial Disease Involving the Vagina, Cervix, and Corpus Uteri." Obstet Gynecol 44, no. 5 (1974): 735-48.
131. Ishihama, A., and T. Makino. "Lymphoid Follicles of the Endometrium in Women Wearing an Intrauterine Device." Am J Obstet Gynecol 107, no. 4 (1970): 535-7.
132. Sen, D. K., and H. Fox. "The Lymphoid Tissue of the Endometrium." Gynaecologia 163, no. 6 (1967): 371-8.
